# Time to Treatment and Risk Factors for Unsuccessful Treatment Outcomes among People Who Started Second-Line Treatment for Rifampicin-Resistant or Multi-Drug-Resistant Tuberculosis in the Kyrgyz Republic, 2021

**DOI:** 10.3390/tropicalmed8080407

**Published:** 2023-08-10

**Authors:** Bolot Kyrbashov, Aizat Kulzhabaeva, Abdullaat Kadyrov, Atyrkul Toktogonova, Collins Timire, Srinath Satyanarayana, Kylychbek Istamov

**Affiliations:** 1 National Center for Phthisiology, Bishkek 720020, Kyrgyzstan; abdylat.kadyrov@gmail.com (A.K.); atyrkul7@gmail.com (A.T.); 2 Public Foundation KNCV-KG, Bishkek 720000, Kyrgyzstan; a.kulzhabaeva@list.ru; 3 Public Health Department, Kyrgyz State Medical Academy, Bishkek 720020, Kyrgyzstan; 4 International Union against Tuberculosis and Lung Disease, 2 Rue Jean Lantier, 75001 Paris, France; collinstimire2005@yahoo.com (C.T.); ssrinath@theunion.org (S.S.); 5 School of Medicine, Osh State University, Osh City 723500, Kyrgyzstan; istamovk@gmail.com

**Keywords:** SORT-IT, RR-TB, Kyrgyzstan, Central Asia, drug resistant tuberculosis

## Abstract

The Kyrgyz Republic is a high-burden country for rifampicin resistant/multi-drug resistant tuberculosis (RR/MDR-TB). TB control efforts rely on early diagnosis and initiation of people on effective regimens. We studied the interval from diagnosis of RR-TB to starting treatment and risk factors for unsuccessful outcomes among people who started RR/MDR-TB treatment in 2021. We conducted a cohort study using country-wide programme data and used binomial regression to determine associations between unsuccessful outcomes and predictor variables. Of the 535 people included in the study, three-quarters were in the age category 18–59 years, and 68% had past history of TB. The median (IQR) time from onset of TB symptoms to diagnosis was 30 (11–62) days, 1 (0–4) days from diagnosis to starting treatment, and 35 (24–65) days from starting treatment to receipt of second-line drug susceptibility test (SL-DST) results. Overall, 136 (25%) had unsuccessful outcomes. Risk factors for unsuccessful outcomes were being homeless, fluroquinolone resistance, having unknown HIV status, past TB treatment, male gender and being unemployed. Treatment outcomes and the interval from diagnosis to starting treatment were commendable. Further reductions in unsuccessful outcomes by be achieved through ensuring timely diagnosis and access to SL-DSTs and by reducing the proportion of people who are lost to follow-up.

## 1. Introduction

Tuberculosis (TB) is a global public health challenge and, until the coronavirus, was the leading cause of death from a single infectious disease (ranking above HIV/AIDS). An estimated 10.6 million people fell ill with TB globally in 2021, and 1.6 million (15.1%) of them died [1]. TB treatment success (treatment completion and cure) are worse among people treated for rifampicin-resistant or multidrug-resistant TB (RR/MDR-TB). Of the global cohort of 185,448 people who started RR/MDR-TB and pre-XDR treatment in 2019, 60% of them attained successful outcomes [1]. Treatment outcomes are slightly lower in the WHO European Region where Kyrgyz Republic lies, at 57%. RR/MDR-TB is expensive to treat, costing approximately USD 5659 per patient [1]. It aggravates poverty at a household level and is usually associated with high proportions of deaths and loss to follow-up. People who are lost to follow-up may be foci for community transmission of drug-resistant TB strains.

The Kyrgyz Republic, with an estimated incidence of RR/MDR-TB of 49 per 100,000 population, is among the 30 countries with a high-burden of RR/MDR-TB [1]. The Kyrgyz Republic’s TB control efforts are a mix of biomedical and action on social determinants of TB as espoused in the End TB strategy [2]. Biomedical approaches include active TB case finding and use of new regimens such as bedaquiline, pretomanid and linezolid (BPaL). In December 2022, the WHO recommended a short 6 month regimen comprising bedaquiline, pretomanid, linezolid and moxifloxacin (BPaLM) but it is yet to be rolled out in most settings [3,4]. Studies have reported a higher treatment success among people on shorter regimens in the Kyrgyz Republic [5]. Recent improvements in treatment success from 62–72% treatment in the 2019 cohort of RR/MDR-TB patients could be attributed to the use of shorter regimens [1]. However, shorter regimens are just one piece of a puzzle in ensuring successful outcomes.

Resistance to fluoroquinolones (FQs) is associated with unsuccessful outcomes among people on RR/MDR-TB treatment [6,7]. Access to timely second-line DST results, especially for FQs, is therefore crucial in contexts such as the Kyrgyz Republic, where resistance to FQs may be much higher than the estimated 18% due to widespread and irrational use of FQs in the country [8,9]. This may inform timely clinical decisions regarding effective regimens. However, according to the Global TB Report 2022, around 30% of people in the Kyrgyz Republic who were diagnosed with RR/MDR-TB did not access DST results for FQ in 2021 [1]. The proportion could be higher among people who live in regions that are far away from the reference laboratory. Sputum specimen transportation challenges have reduced access to DST results, especially in peripheral areas in other settings [10,11].

TB has biomedical and socioeconomic dimensions. Hence, the role of social factors in influencing unsuccessful outcomes cannot be ignored. Studies have reported poorer outcomes among key populations (e.g., migrants, the homeless, the socially vulnerable, HIV infected and ex-prisoners) [5,12,13]. While there have been improvements in treatment outcomes in the Kyrgyz Republic, the impact of COVID-19, delayed health seeking behaviour and drug toxicities may also affect outcomes. As per the TB programme reports, 28% of people who started treatment in 2019 had unsuccessful outcomes. However, the factors associated with unsuccessful outcomes are unknown. If this knowledge gap is filled, targeted interventions may be introduced to improve treatment outcomes in this population. A PubMed search revealed scant literature on risk factors for RR/MDR-TB treatment from the Kyrgyz Republic. We aimed to describe the demographic, clinical profiles, treatment outcomes and factors associated with unsuccessful outcomes among people who started second-line drug regimens for RR/MDR-TB treatment in the Kyrgyz Republic in 2021. We also aimed to describe the turnaround times from onset of TB symptoms to diagnosis, starting treatment and to receipt of second-line DST results.

## 2. Materials and Methods

### 2.1. Study Design

Cohort study using programme data.

### 2.2. Study Setting

#### 2.2.1. General Setting

The Kyrgyz Republic is a middle-income country with an area of 198,500 km^2^ and a population of 6,747,300 people [14]. The country shares borders with Kazakhstan, Tajikistan, Uzbekistan and China. There are seven regions and two large cities.

The proportion of RR/MDR-TB cases is 27% among new cases and 59% among previously treated TB cases [15]. There are 26 Gene Xpert machines, mostly placed within primary health care centres throughout the country. These centres refer specimens twice a week to the national reference laboratory for genotypic and phenotypic second-line DST. A national specimen transportation system run by a courier company transports specimens from all regions to the reference laboratory. The system is mostly funded by the country’s mandatory health insurance fund and partly by international partners.

TB treatment is provided free of charge. Patients receiving treatment as outpatients have to be registered with the Family Medicine Center in order for them to benefit from a package of free medical services. They also have the right to apply to local authorities for material assistance.

#### 2.2.2. Specific Setting

##### Laboratory Diagnosis of RR-TB

All people who are presumptive of TB are tested for TB using smear microscopy and Xpert MTB/RIF assay (Cepheid, Sunnyvale, CA, USA), as per national guidelines. Once resistance to rifampicin is detected, a patient is started on second-line treatment. At that time, a second sputum specimen is sent to the national reference laboratory for both culture and phenotypic DST on MGIT and Lowenstein Jensen (LJ) media, and genotypic first-line and second-line DST using Hain Line Probe Assay (Hain LifeSciences, GmbH, Nehren, Germany).

#### 2.2.3. Management of RR-TB in the Kyrgyz Republic

All patients with RR-TB are immediately started on the SHORRT regimen 4–6 (Bdq-Mfx-Cfz-E-H-(HD)-Pto/5 (Mfx-Cfz-E-Pto) pending DST results. Treatment regimen is decided on a case-by-case basis by DR-TB Consilia. The Consilia determine the composition of regimens, including number of drugs that are considered effective to maximize treatment success, reduce treatment failure and adverse drug events (ADEs). During the study period, there were no reports of stock outs of medicines to treat RR-TB. Patients in whom FQ resistance is presumed are started on a bedaquiline, pretomanid and linezolid (BPaL) regimen and, upon receiving second-line DST results, may be switched to individual regimens as shown in Table 1 below.

Patients are managed by either video directly observed therapy (VDOT), or family or facility DOT. They undergo regular clinical and laboratory tests to monitor both effectiveness of treatment and potential ADEs. Drugs that lead to serious ADEs are stopped and data on ADE are reported systematically using appropriate reporting forms (yellow forms).

Patient level data are recorded in three interlinked electronic management systems: the Laboratory Management Information System, the electronic TB register and the electronic medical card. This makes it possible for requesting clinicians to access RR-TB results as soon as they are uploaded by laboratories. Treatment outcomes are documented as per WHO guidelines, as shown in Table 2.

#### 2.2.4. Social Protection for People on RR/MDR-TB Treatment in the Kyrgyz Republic

Some patients in pilot sites who are on ambulatory treatment receive USD 12 monthly, which is transferred to their bank accounts. The money is meant to cover expenses on transport and nutrition, and is conditional on patients adhering to scheduled visits. The Red Cross Society provides monthly food packages to all vulnerable TB patients including migrants, ex-prisoners, HIV co-infected, low-income people and the homeless. People with TB are cushioned against transport costs to medical facilities using local funds.

### 2.3. Study Population and Period

The study population is all patients who started second-line drug regimens for RR/MDR-TB and XDR treatment in Kyrgyz Republic from January to December 2021.

### 2.4. Data Variables, Sources of Data and Data Collection

The following variables were collected for the purposes of the study. Age in years; sex; migrant status; comorbidities (HIV, diabetes status); alcohol use; smoking status; drug use status; homeless status; employment status; type of TB (new, previously treated); region (Bishkek, Osh, etc.); place of residency (rural/urban); whether sputum specimen was sent to reference laboratory (Yes/No); whether second-line DST was performed (Yes/No); fluoroquinolone resistance profiles; dates of (i) onset of TB symptoms, (ii) TB diagnosis, (iii) TB treatment initiation, (iv) receipt of second-line DST results; and outcomes of RR/MDR and XDR-TB treatment (cured, completed treatment, died etc.). Data sources were laboratory management information system (LMIS), the electronic TB register and electronic medical card.

#### Data Collection and Validation

Patient-level data were entered into various electronic databases as a part of routine work. Each patient record had a unique identifier that was used to track the patient in the three databases. The censor date for ascertaining outcomes was 28 February 2023. Electronic data were exported from various databases to MS Excel. Once in Excel, data were cleaned to remove duplicates and fuzzy lookup was used to merge the three Excel databases into a single database comprising patient and laboratory data.

### 2.5. Data Analysis and Statistics

Patient-level data were cleaned in MS Excel and were imported into Stata v13 (StataCorp, College Station, TX, USA) for further cleaning and analysis. A new binary variable for treatment outcome (success or unsuccessful) was created. Categorical variables were summarised using frequencies and percentages. Continuous variables were assessed for normality using the Shapiro–Wilk test. Skewed data were presented as medians and interquartile ranges (IQRs). Time taken to receive second-line DST results were obtained by subtracting date of starting treatment from date of receiving DST results and were presented as medians and IQRs. We used the median cut-off of one day for time to start standardized second-line treatment to create a binary variable indicating either early or delayed treatment. Binomial regression was then used to test the association between unsuccessful outcomes and sociodemographic and clinical factors and results were presented as relative risks (RRs) and 95% confidence intervals. Level of significance was set at *p* ≤ 0.05.

## 3. Results

### 3.1. Sociodemographic Characteristics of Study Participants

The sociodemographic characteristics of the 535 people with RR/MDR-TB who were enrolled in the study are presented in Table 3. Almost 60% were males and 75% of them were in the age categories 18–59 years. The mean (SD) age was 38.5 (17.8) years. One third were previously treated of TB, and just above a quarter had no documented HIV status. Almost 80% were from the regions Chui, Bishkek, Jalal-Abad and Osh Oblast. Less than 10% reported alcohol use, smoking and diabetes.

### 3.2. Intervals from Onset of TB Symptoms to Diagnosis and Treatment Initiation

Of the 496 people with valid dates, the median time from onset of TB symptoms to TB diagnosis was 30 days. Migrants and homeless people had median times (IQR) of 35 days (21–58) and 56 days (16–176), respectively. There was no difference in diagnostic delays by history of TB, *p* = 0.99.

Overall, 256 (55%) people started treatment within a day of RR/MDR-TB diagnosis, while 75% started treatment within 4 days of RR/MDR-TB diagnosis. Using the median turnaround time of one day as a cut-off, 55% of the people received treatment within a day. Half of the people received second-line DST results within 35 days, as shown in Table 4.

### 3.3. Treatment Outcomes of People Who Started RR/MDR-TB Treatment

Treatment outcomes are shown in Table 5 below. Overall, 25% of the people had unsuccessful outcomes. People who were either not evaluated or LTFU accounted for around 50% of the unsuccessful outcomes.

### 3.4. Factors Associated with Unsuccessful Outcomes

The factors associated with unsuccessful outcomes are presented in Table 6. Overall, 136 (25%) (95%CI: 22–29%) had unsuccessful outcomes. Risk of unsuccessful outcomes was high among males, the homeless, the unemployed, migrants, people who were previously treated for TB, people with undocumented or unknown HIV status and people who had resistance to fluroquinolones (FQs). Males had a 42% higher risk of unsuccessful outcomes compared to females. Risk of unsuccessful outcomes was 2.1 times higher in people with FQ resistant strains, (relative risk (RR) = [2.09. 95%CI: 1.54–2.84]), and was much higher in homeless people, RR = 2.87 [2.09. 95%CI: 1.76–4.71]). Compared to people with a negative HIV status, those with undocumented HIV status were 2.7 times more likely to have unsuccessful outcomes, RR = [2.66. 96%CI: 2.00–3.55]). There were no differences in risk of unsuccessful outcomes by variables such as place of residency and migrant status.

## 4. Discussion

In this study we found longer time intervals from onset of TB symptoms to RR/MDR-TB diagnosis and from starting treatment to accessing second-line DST results, but shorter intervals between diagnosis of RR-TB and starting treatment. There were huge gaps in documenting HIV status. The proportion of unsuccessful outcomes was remarkably low and risk factors for unsuccessful outcomes were being homeless, FQ resistance, having unknown HIV status, past TB treatment, male gender and being unemployed.

A previous study on prison inmates in the Kyrgyz Republic reported median delays from diagnosis to starting treatment of 7 days and further delays in people with RR-TB [17]. In other settings, despite the introduction of Xpert MTB/Rif technology, median treatment delay ranged from 8 days in Ethiopia to 11 and 14 days in South Africa [18,19,20]. This study therefore reports a remarkably shorter interval from diagnosis to starting treatment. However, some people experienced delays of up to 20 days. While it was good to start people on treatment early, it is crucial to understand reasons for such delays. The TB program has provided access to rapid diagnostic methods recommended by WHO, and councils that prescribe treatment have been decentralized and are accessible in each region. Longer delays in starting treatment are mainly due to patients who belong to the category of migrants and the homeless.

There were marked delays among the homeless and migrants. Reasons for delays in being diagnosed with RR-TB (diagnostic delays) may be multifactorial. On the one hand, diagnostic delays may be due to person related factors such as delayed/poor health-seeking behaviour. People may delay health-seeking when they think the illness is due to other causes [21] or when they perceive the quality of services in public facilities to be low and they initially seek healthcare from pharmacies and private healthcare providers [22,23]. These institutions may have low indices of suspicion for TB leading to missed opportunities to diagnose RR-TB. Thus, passive case finding strategies that allow people to visit health facilities may not detect all cases of TB. Qualitative interviews from Africa revealed that people who experienced TB were quick to identify TB symptoms and to seek TB services from public health facilities early [24]. This averts physical debility and community transmission of TB. However, we did not observe this in our study. On the other hand, delays may be due to health-system-related factors. This may occur if diagnostic tests such as smear microscopy or when culture methods are used to diagnose TB. While the former is less sensitive and cannot diagnose RR-TB, the latter takes a long time before a diagnosis is made. The World Health Organisation (WHO) approved rapid diagnostic technology (WRD) such as Xpert MTB/Rif (Ultra) (Cepheid, Sunnyvale, CA, USA) to offer rapid diagnosis of TB, including resistance profiles for rifampicin. Yet, the WHO reports that around 25% of notified TB cases in the Kyrgyz Republic were not diagnosed using WRD [15]. Delays in starting treatment increase the risk of unsuccessful treatment outcomes and community transmission of RR-TB. The scaling up of WRDs and ensuring their effective utilisation may increase the proportion of people who are diagnosed with these instruments. This may reduce intervals from onset of symptoms to diagnosis. This will require efficient sputum specimen transportation and referral systems to Gene Xpert centres [10,11,25].

FQ resistance is one of the risk factors for unsuccessful outcomes [6,7]. In this study, we observed long median turnaround times of up to 35 days to accessing second-line DSTs. The delays, therefore, imply that the standardised regimen was suboptimal therapy for a long time in around 15% of patients in whom FQ resistance was detected. Suboptimal therapy increase the risk of community transmission of drug-resistant TB strains, morbidity, and mortality and may increase the risk of pre-XDR and XDR-TB strains [26,27]. To ensure better treatment outcomes, people must be started on effective regimens. This requires early access to DSTs to inform clinical decisions regarding type and mix of medicines to be included in individualised regimens [27,28]. A short, 6 month all-oral BPaLM regimen was recommended by the WHO in December 2022. This is a game changer in treating RR-TB, even in people with additional resistance to FQ (pre-XDR-TB) [3,4]. The regimen has a shorter duration, safety profile and better treatment outcomes than standard 9–12 month long regimens.

HIV is one of the risk factors for TB and TB/HIV co-infection is associated with poor treatment outcomes. The high proportion of undocumented HIV status is worrisome and has been reported previously [5]. Though the TB/HIV co-infection of 4% is markedly lower than figure of at least 50% reported in sub-Saharan Africa [1]. HIV testing is key to ensure linkage to ART care, reduce morbidity and to improve treatment outcomes. However, even among those people with TB who had known positive HIV status, up to 40% were not started on antiretroviral therapy (ART) around 2021 [15].

Several studies have documented treatment outcomes in the Kyrgyz Republic that range from 50% to 83%, depending on type of regimen [5]. The proportion of unsuccessful treatment outcomes observed in this study is comparable to the proportion 28% for the Kyrgyz Republic, which was reported in the global TB report of 2022, but is remarkably lower than the global figure of around 40% [1]. The outcomes are commendable, and reasons for reductions in unsuccessful outcomes in the Kyrgyz Republic could be attributed to a combination of factors including transitioning to shorter regimens including BPAL and BPaL(M) [5], complementing biomedically oriented TB control efforts with social protection to support people on TB treatment so they complete treatment. Successful outcomes of up to 83% were reported in a cohort of people who started short 9–12 month regimens in the Kyrgyz Republic [5]. This informed the scaling up of shorter regimens.

Our study is strengthened by the fact that we used nationwide programme data for all people who started on treatment in 2021. As a result, our findings reflect the situation on the ground and were free from selection bias.

We acknowledge some limitations. Firstly, as with most programme data, we had some missing data. Secondly, we could not calculate delays in starting individualised treatment since dates of starting individualised treatment were not captured. Thirdly, the data we had made it difficult to tease out the people who were switched to individualised regimens. This made it difficult to disaggregate analysis by type of regimen. Shorter regimens are associated with successful outcomes as compared to standard of care [3,5]. Thirdly, we could not disaggregate analysis by type of regimen, as it was difficult to tease out the people who were switched to individualised regimens. Lastly, we could not assess the protective effects of social protection against unsuccessful outcomes. There is a dearth of studies that investigated the effectiveness of social protection on RR-TB treatment outcomes. Most studies focused on people with DS-TB and have shown that social protection improves treatment outcomes [29,30,31]. Hence, we missed an opportunity to contribute to knowledge regarding the effectiveness of social protection among people with RR-TB.

Despite these limitations, our results have important considerations for both policy and practice. Firstly, delays in receiving diagnoses of RR-TB may be reduced by increasing awareness about TB symptoms within communities and the benefits of early health seeking for TB, the use of TB self-check apps and enhanced collaborations between the public sector and the private sector. Public–private partnerships may increase case notifications by tapping into people who first visit private facilities. Secondly, there is need to strengthen collaborations for TB/HIV and other comorbidities including diabetes mellitus. This ensures that those diagnosed with the comorbidities are linked to care to improve treatment outcomes. Both HIV and diabetes are risk factors for TB, and are responsible for worse outcomes as observed in this study and elsewhere. Thirdly, the long turnaround time for DSTs, including FQ, implies that people take suboptimal therapies for a long period before they are switched to effective and individualised therapies. From an operational point of view, there is need to improve efficiency of existing systems to shorten the time to accessing second-line DSTs. The TB programme may also consider investing in point-of-care rapid diagnostic technology such as the Gene Xpert MTB/XDR (Cepheid, Sunnyvale, CA, USA) that offers resistance profiles for isoniazid, rifampicin, FQs and second-line injectables [32,33]. This assay is a game changer since it is deployable to peripheral areas to ensure faster, near-patient and enhanced access to second-line DSTs.

## 5. Conclusions

Both treatment outcomes and the interval from diagnosis to starting treatment were commendable. Further reductions in unsuccessful outcomes in the Kyrgyz Republic are possible through ensuring timely diagnosis and access to SL-DSTs and by reducing the proportion of people who are not evaluated or lost to follow-up.

## Figures and Tables

**Table 1:** RR/MDR-TB treatment regimens and their durations in the Kyrgyz Republic, 2021.

Types of Regimens	Dosage and Duration of Treatment
SHORRT regimens	4–6 Bdq-Mfx-Cfz-Lzd(2)-E-Z-Hhd/5 Mfx-Cfz-E-Z
BPaL	6–9 Bdq-Lzd–Pa
Individual regimens	18–20 Lfx-Bdq-Lzd-Cfz-Cs 18–20 Lfx-Bdq-Dlm-Cfz-Cs 6 Bdq-Lzd-Cfz-Dlm-Cs/12 Bdq-Lzd-Cfz-Cs

Bdq = bedaquiline; E = ethambutol; H = isoniazid; Pa = pretomanid; Lzd = linezolid; Cs = cycloserine; Dlm = delamanid; Cfz = clofazimine; BPaL = bedaquiline, pretomanid and linezolid.

**Table 2:** Outcomes for RR/MDR-TB/XDR-TB patients treated using second-line treatment.

Outcome	Definition
Cured	A pulmonary TB patient with bacteriologically confirmed TB at the beginning of treatment who completed treatment as recommended by the national policy with evidence of bacteriological response and no evidence of failure.
Treatment completed	A patient who completed treatment as recommended by the national policy whose outcome does not meet the definition for cure or treatment failure.
Treatment failed	A patient whose treatment regimen needed to be terminated or permanently changed to a new regimen or treatment strategy.
Died	A patient who died before starting treatment or during the course of treatment.
Lost to follow-up	A patient who did not start treatment or whose treatment was interrupted for 2 consecutive months or more.
Not evaluated	A patient for whom no treatment outcome was assigned.
Treatment success	The sum of cured and treatment completed.

Source: Linh et al., 2021 [16].

**Table 3:** Sociodemographic characteristics among people who started second-line drug regimens for RR/MDR-TB and XDR treatment in Kyrgyz Republic, 2021.

Characteristics		n	(%) †
Total		535	
Sex	Male	310	(58)
	Female	225	(42)
Age group (years)	<15	23	(4)
	15–17	20	(4)
	18–44	300	(56)
	45–59	110	(21)
	≥60	82	(15)
Region	Chui	138	(26)
	Jalal-Abad	113	(21)
	Bishkek city	91	(17)
	Osh oblast	74	(14)
	Issyk Kul	21	(5)
	Osh city	30	(6)
	Talas	25	(5)
	Batken	22	(4)
	Naryn	21	(4)
Residency	Rural	302	(57)
	Urban	226	(42)
	Not recorded	7	(1)
Risk groups	Homeless	7	(1)
	Migrant	40	(7)
	Employed	236	(44)
	Diabetes mellitus	57	(11)
	Drug use	2	(<1)
	Alcohol use	31	(6)
	Smoking	46	(9)
HIV status	Positive	21	(4)
	Negative	364	(68)
	Unknown	150	(28)
Type of TB case	Previously treated	170	(32)
	New	365	(68)

† = column percentages; HIV = human immunodeficiency virus.

**Table 4:** Intervals from onset of TB symptoms to diagnosis, treatment initiation and accessing second-line DST results among people who started second-line drug regimens in the Kyrgyz Republic, 2021.

Duration of Care		Number of People with Valid Dates	Median (IQR) *
TB * symptoms–TB diagnosis		496	30 (11–62)
TB diagnosis–Start of standardised TB treatment		465	1 (0–4)
Start of standardised treatment–receipt of SL-DSTs *		261	35 (24–65)
Started treatment ⁑	Same day	197 (42%)	
<1 day		59 (13%)	
<2 days		48 (10%)	
<3 days		29 (6%)	
<4 days		19 (4%)	

⁑ = Standardised treatment; * TB—tuberculosis; * SL-DST––second-line drug sensitivity test, * IQR–interquartile range.

**Table 5:** Treatment outcomes of among people who started second-line drug regimens for RR/MDR-TB and pre-XDR treatment in the Kyrgyz Republic, 2021.

Treatment Outcomes		n	(%)
Successful outcome		399	(75) †
	Cured	287	(54)
	Treatment completed	112	(21)
Unsuccessful outcome		136	(25)
	Treatment Failure	25	(5)
	Died	44	(8)
	Not evaluated/LTFU	67	(12)

† = Column percentages; LTFU = loss to follow-up.

**Table 6:** Factors associated with unsuccessful outcomes among people who started second-line drug regimens for RR/MDR-TB and pre-XDR treatment in the Kyrgyz Republic, 2021.

Factors		Total	Unsuccessful Outcomes		RR	(95% CI)
			n	(%) ‡		
Total		535	136	(25)		
Age in years	<15	23	2	(9)	0.32	(0.10–1.42)
	15–17	20	0	(0)	-	-
	18–44	300	70	(23)		Ref
	45–59	110	38	(35)	1.48	(1.07–2.06)
	≥60	82	26	(32)	1.36	(0.93–1.98)
Sex	Male	310	90	(29)	1.42	(1.04–1.94)
	Female	225	46	(20)		Ref
Region	Chui	138	39	(28)	1.71	(1.01–2.92)
	Jalal-Abad	112	31	(28)	1.68	(0.97–2.91)
	Bishkek city	91	15	(15)		Ref
	Osh oblast	74	17	(23)	1.39	(0.75–2.60)
	Osh city	30	12	(40)	2.43	(1.28–4.59)
	Other *	90	22	(24)	1.48	(0.82–2.67)
Residency	Rural	302	77	(26)	1.02	(0.76–1.37)
	Urban	226	59	(26)		Ref
Homeless	Yes	7	5	(71)	2.87	(1.76–4.71)
	No	528	131	(25)		Ref
Employed	es	236	45	(19)		Ref
	No	299	91	(30)	1.59	(1.17–2.18)
Migrant	Yes	40	10	(25)	1.01	(0.84–1.21)
	No	495	126	(25)		Ref
HIV status	Positive	21	6	(29)	1.67	(0.82–3.42)
	Negative	364	62	(17)		Ref
	Not recorded	150	68	(45)	2.66	(2.00–3.55)
Type of TB	New	365	77	(21)		Ref
	Previously treated	170	59	(35)	1.65	(1.24–2.19)
FQ resistance	Yes	76	34	(45)	2.09	(1.54–2.84)
	No	444	95	(21)		Ref
Delayed treatment ∮	Yes	209	58	(28)	1.18	(0.87–1.62)
	No	256	60	(23)		Ref

‡ = Row percentages; TB—tuberculosis; HIV—human immunodeficiency virus; FQ = Fluoroquinolone; TB—tuberculosis; PTB—pulmonary TB; HIV—human immunodeficiency virus; RR—risk ratio; aRR—adjusted risk ratio; CI—confidence interval. ∮ = delay from diagnosis and treatment in days, classified by median cut-off of 1 day; * includes Talas, Batken, Issy-Kul and Naryn.

## Data Availability

Requests to access these data should be sent to the corresponding author: bolotkyrbashov@gmail.com (B.K).
